# The Current State of MPOWER Policies in the Republic of Kazakhstan: Data from the Global Adult Tobacco Survey 2014

**Published:** 2019-05

**Authors:** Shynar ABDRAKHMANOVA, Zarina KERUYENOVA

**Affiliations:** National Center of Public Health, Nur-Sultan, Kazakhstan

**Keywords:** Tobacco use, Global adult tobacco survey, Kazakhstan

## Abstract

**Background:**

The Global Adult Tobacco Survey (GATS) is a global study to monitor tobacco use, and evaluate current measures and requisite policies on tobacco control. In this study, the key indicators from GATS Kazakhstan that address six tobacco control policies are assessed.

**Methods:**

GATS Kazakhstan was a nationally representative household survey of the adult population aged 15 yr or older (n=4425, 52.8% females) implemented in 2014, in all regions of Kazakhstan. A multi-stage, geographically clustered sample design was used to obtain the key indicators of tobacco use and tobacco measures in the country. The sampling weights were employed to ensure real national representation. A household and individual questionnaire were administered through electronic devices.

**Results:**

The majority of current tobacco users were cigarette smokers 22.2%. Overall, 19.0% of adults were exposed to secondhand smoke while at work. One third of smokers made a quit attempt in the last 12 months. Only, 74.0% of adults believed that breathing other people’s smoke causes serious illness in non-smokers. Almost all current smokers (97.6%) noticed pictorial health warnings on cigarette packages. Over half (58.0%) of the current smokers had thought about quitting, having seen pictorial warning labels. Exposure to any cigarette advertisement, sponsorship or promotion had been experienced by 25.7% of adults, with the highest rate of noticing cigarette advertisements being found in stores where cigarettes are sold (14.0%). Cigarettes were largely affordable for the population.

**Conclusion:**

The GATS Kazakhstan results identified tobacco use indicators, as well as existing gaps in tobacco control measures.

## Introduction

Tobacco use is the greatest cause of preventable death globally and causes 6 million deaths annually worldwide (1,2). The adverse human, social, and economic consequences of tobacco use impose burdens for all countries, but these impacts are particularly high in low- and middle-income countries (3). By the year 2030, more than 80% of tobacco-related deaths will occur in developing countries (4). On May 21, 2003, in response to the devastating tobacco epidemic, the WHO Framework Convention on Tobacco Control (WHO FCTC) was developed, and on Feb 27, 2005, it was enforced as the first international treaty that specifically aimed to protect people through proven tobacco control measures.

In 2008, to support countries in fulfilling the obligations of WHO FCTC, WHO established MPOWER, a package of six proven tobacco control policy measures that aimed to reverse the global tobacco epidemic (4–6). The MPOWER package includes **M-**monitoring tobacco use and prevention policies; **P-**protecting people from tobacco smoke; **O-**offering help in quitting tobacco use; **W-**Warning about the dangers of tobacco; **E-**enforcement of tobacco advertising, promotion, and sponsorship bans; and **R-**raising taxes on tobacco products (4).

Kazakhstan signed the FCTC in 2004, and it was ratified by the Low “On ratification of the WHO Framework Convention on Tobacco Control” in November 2006 (7). Since then, Kazakhstan’s legislation has made remarkable progress in terms of tobacco demand reduction and has carried out the country’s international obligations in implementing tobacco control measures to protect citizens and improve the health of the population. In 2014, Kazakhstan implemented the Global Adult Tobacco Survey (GATS), in order to monitor tobacco use and to obtain internationally comparable data as well as evaluate current measures and requisite policy on tobacco control. GATS is a systematic surveillance tool for the effective evaluation and analysis of all six MPOWER measures and subsequently allows for the FCTC articles on tobacco demand and supply to be addressed.

In the study, we used key indicators from GATS for each of the 6MPOWER measures to assess the status of tobacco control policy in Kazakhstan.

## Materials and Methods

GATS Kazakhstan was a nationally representative household survey of the adult population. GATS uses a standard protocol and a core questionnaire with a set of optional questions (8). In the survey, a stratified three-stage cluster sample design was used to produce nationally representative data. The primary sampling units were settlements consisting of households in rural and urban areas. The second stage of selection were residential addresses from the National Housing Registry. In the final stage, one individual is randomly selected from each participating household. The target population included all households in the country and residents of the households aged 15 yr and older.

The active consent approach was used to obtain approval from respondents and their parent/guardian as appropriate. The survey protocol got approval from local Ethical Committee.

Institutionalized public settings and facilities designed for rest or temporary accommodation were excluded from the sample. Two types of questionnaires were administered: household and individual ones. The questionnaires were based on the GATS Core Questionnaire with Optional Questions. The household questionnaire collected information about all adult residents to select randomly an eligible respondent for individual interview. The individual questionnaire had the following sections: background characteristics; tobacco use (smoked and smokeless tobacco, e-cigarettes; cessation; secondhand smoke; economics; the media; knowledge, attitudes, and perceptions; pictorial health warnings. The questionnaires were adapted, translated and back translated to ensure the accuracy of translation.

The CDC Questionnaire Review Committee approved the questionnaires. The pre-test was conducted in Feb–Mar 2014. The final version of the questionnaires were approved and uploaded into handheld devices. The field supervisors and field workers were trained on Feb 10 to 14, 2014 on the survey methodology, use of electronic data collection devices, data transfer. Mock interviews were performed. The fieldwork was conducted Apr 2–30, 2014.

Prior to data analysis, the data were weighted to the national adult population by calculating the sample weights for each respondent. The complex survey analysis was performed using SPSS (ver.21, Chicago, IL, USA) and SAS version 9.3 to obtain prevalence rates as percentages with a 95% confidence interval (CI). Standard errors were calculated using Taylor series linearization. Tests of statistical differences were performed by comparing the 95% CIs of the two estimates.

The GATS indicators that correspond to implementation of tobacco control measures (monitoring, exposure, demand, reduction) are presented in the article. There are six groups of indicators (9):

1. Overall and gender-specific current tobacco use, current cigarette smoking, current smokeless tobacco use.
2. Exposure to tobacco smoke at indoor workplaces and in other public spaces in the last 30 d.
3. Tobacco cessation: attempts to quit smoking in the past 12 months, health care professionals advising to quit smoking during medical consultation in the past 12 months; interest in quitting smoking.
4. Awareness about the dangers of tobacco: adults noticed anti-cigarette smoking information; noticed health warning labels and pictorial labels on cigarette packaging; thoughts of quitting due to health warning labels and pictorial labels on cigarette packaging; beliefs about the dangers of smoking and exposure to secondhand smoke.
5. Noticed cigarette marketing during the last 30 d in various places.
6. Economic indicators showing cigarette affordability: average amount spent on 20 manufactured cigarettes; average cigarette expenditure per month; Cost of 2,000 manufactured cigarettes as a percentage of per capita Gross Domestic Product (GDP).

## Results

Of 4611 households in the final sample, 4,451 participated in the survey and 4,425 individuals completed the individual interview. Therefore, the household response rate was 97.2%, the individual response rate was 99.4% and the overall response rate was 96.7%. By gender, there were 47.2% males and 52.8% females, by place of residence- 56.6% urban and 43.4% rural population, by education- 6.0% had primary, 24.0% -secondary education, 30.6%-secondary vocational and 39.3% higher education (the weighted data).

Overall, the current tobacco use prevalence among adults was 22.9%. The majority of current tobacco users were cigarette smokers (22.2%), which represents 2,794,500 people. Cigarette smoking prevalence was far greater among males (42.2%) than females (4.2%). The current smokeless tobacco use rate was low, at 1.3% (Table 1).

**Table 1:** Summary MPOWER indicators, GATS Kazakhstan 2014

	***Overall***	***Gender***	

***Indicator***		***Male***	***Female***
**M: Monitor tobacco use and prevention policies**	Percentage (95% CI)				
Current tobacco use	22.9(21.2, 24.7)	43.4(40.6, 46.3)	4.5(3.5, 5.8)
Current cigarette smokers	22.2 (20.5, 24.0)	42.2(39.4, 45.0)	4.2(3.3, 5.5)
Current smokeless tobacco use	1.3(1.0, 1.8)	2.8(2.0, 3.9)	0
**P: Protect people from tobacco smoke**			
Exposure to secondhand smoke at work^†^	19.0(16.0, 22.5)	24.7(20.7, 29.1)	12.9(9.7,17.1)
Government buildings/offices	9.9(5.7, 13.2)	12.5(9.3, 16.7)	7.8(5.7, 10.5)
Health care facilities	9.7(6.9, 13.5)	11.3(8.0, 15.6)	8.8(5.7, 13.2)
Restaurants	27.6(23.1, 32.6)	32.8(26.9, 39.4)	22.,2(17.8, 27.4)
Public Transportation	18.1(15.2, 21.4)	19.4(15.6, 24.0)	17.2(14.0, 20.9)
**O: Offer help to quit tobacco use**			
Advised to quit smoking by a health care provider	46.6(40.2, 53.1)	49.8(40.2, 53.1)	27.9(15.7, 44.4)
Attempted to quit smoking using a specific cessation method:	29.5(26.3,32.9)	28.9(25.6,32.5)	34.3(25.4–44.5)
Quit without assistance	76.5(69.2, 82.5)	77.0(69.3, 83.2)	72.7(54.8, 85.4)
Pharmacotherapy	23.4(18.0, 29.9)	21.7(16.5, 27.8)	35.7(18.7, 57.3)
Counseling/advice	10.2(6.2, 16.4)	9.3(5.4, 15.3)	17.1(5.1, 44.5)
Interest in quitting smoking	63.9(56.5 72.2)	63.4(56.3 71.5)	67.4(43.6102.9)
**W: Warn about the dangers of tobacco**			
Belief that tobacco smoking causes serious illness	84.9(82.8, 86.7)	79.1(76.1, 81.8)	90.1(88.2, 91.7)
Belief that breathing other peoples’ smoke causes serious illness	74.0(71.0, 76.8)	65.6(61.8, 69.2)	81.5(78.1, 84.4)
Noticed anti-cigarette smoking information at any location^†^	49.5(45.9, 53.2)	47.1(42.9, 51.3)	51.7(42.9, 51.3)
Thinking of quitting because of health warnings on cigarette packages	51.3(47.5, 55.1)	50.8(46.8, 54.8)	55.5(45.1, 65.4)
Thinking of quitting because of pictorial warning label	58.0(54.0, 61.9)	57.9(53.6, 62.1)	58.5(48.9, 67.4)
**E: Enforce bans on tobacco advertising, promotion, and sponsorship**			
Noticed any cigarette advertisement, sponsorship or promotion^†^	25.7(22.5, 29.1)	26.2(22.5, 30.4)	25.2(21.7, 28.9)
Noticed any cigarette marketing in the stores where cigarettes are sold^†^	14.0(11.4, 17.1)	14.6(11.6, 18.3)	13.4(10.8, 16.6)
**R: Raise taxes on tobacco**			
Average manufactured cigarette expenditure per month (Tenge)	4 244.5(3750.8, 4738.1)	4 420.4(3885.2, 4955.5)	2 602.6(2031.3, 3173.9)
Average cost of a pack of 20 manufactured cigarettes (Tenge)	221.4	222.4	207.2

† In the past 30 d

The overall prevalence rate of secondhand smoke exposure in indoor workplaces was 19.0% (males 24.7%, females 12.9%) in the last 30 d. The percentage of adults who visited various public places in the last 30 d and were exposed to tobacco smoke was the highest in restaurants (overall 27.6%, males 32.8%, females 22.2%), followed by public transportation (overall 18.1%, males 19.4%, females 17.2%), government buildings or offices (overall 9.9%, males 12.5%, females 7.8%), and health care facilities (overall 9.7%, males 11.3%, females 8.8%).

Those Kazakhstan current smokers and former smokers abstained for less than 12 months, and visited health care providers, were asked in GATS if their health care provider had asked them about their smoking status and if the health care provider had advised them to quit. The majority (59.0%) of smokers were asked by their health care professional about smoking during a consultation in the past 12 months about smoking, and 46.6% were advised to quit smoking.

In terms of cessation method used, amongst current smokers and former smokers abstained for less than 12 months, 76.5% had attempted to quit without assistance, 10.2% used counseling and advise, and 23.4% used pharmacotherapy. More than half of the current smokers (63.9%) were interested in quitting smoking.

According to the GATS Kazakhstan 2014 data, 84.9% of all adults believed that tobacco smoking causes serious illness (males 79.1%, females 90.1%). Overall, 74.0% of adults (males 65.6%, females 81.5%) believed that breathing other people’s smoke causes serious illness in non-smokers. For current smokers, 73.0% (68.6% males, 76.9% of females) believed that tobacco smoking causes serious illness, and only 57.3% were aware of health risks of secondhand smoke.

Anti-cigarette smoking information at any location in the last 30 d was noticed by 49.5% of adults (males 47.1%, females 51.7%). The three major channels of antismoking information were television (with 34.6% adults noticing messages on this medium), newspapers or magazines (29.9%) and billboards (20.7%).

Nearly all of the current smokers (97.6%) noticed pictorial health warnings on cigarette packaging. A majority (58.0%) of current smokers thought about quitting because of pictorial warning labels that they had encountered.

According to the GATS results, 25.7% of adults in Kazakhstan were exposed to any cigarette advertisement, sponsorship or promotion (male 26.2%, female 25.2 %). Among various places that advertisements could be, the highest rate of noticed cigarette advertisements in the last 30 d was found to be in stores where cigarettes are sold, at an overall rate of 14.0%.

The average manufactured cigarette expenditure per month 23.30 USD overall, with significant difference seen in expenditure between males (24.30 USD) and females (14.30 USD). The average price paid for a pack of 20 manufactured cigarettes was 1.22 USD. The cost of 100 packs (2,000 cigarettes) as a percentage of per capita GDP for 2014 was 1.0%.

## Discussion

Implementation of tobacco surveys is essential for countries to track and support tobacco control measures and policies. Monitoring programs have to include indicators of different forms of tobacco use, the impact of policy interventions, as well as tobacco industry marketing practices. Article 20 of FCTC calls for surveillance of tobacco consumption indicators and the need for comparable data.

In Kazakhstan, the monitoring of tobacco use has been conducted through periodic national health behavior surveys. 26.5% of adults smoked cigarettes (41.5% males, 11.0% females) (10). However, the differences in methodology do not allow for inter-country comparisons of tobacco use and monitoring of key MPOWER indicators. The Global Youth Tobacco Survey was conducted in 2004, 2009, and 2014. GATS Kazakhstan 2014 provides comprehensive national estimates for monitoring the tobacco epidemic. Tobacco use rates were lower among women than among men. The majority of tobacco users were smokers of manufactured cigarettes. Smokeless tobacco use was relatively low.

Secondhand smoke is responsible for over 600,000 deaths (170,000 of these deaths occur among children) annually (2,11). The implementation of completely smoke-free workplaces and public spaces are the measures that protect non-smokers and encourage smokers to quit (4,5). Article 8 of FCTC addresses the adoption and implementation of effective legislation, as well as administrative and enforcement measures to protect people from tobacco smoke in workplaces, on public transport, and in public places.

The Republic of Kazakhstan has banned smoking in indoor workplaces, government buildings or offices, health care and education facilities, public transportation, and other public places. However, designated smoking areas are allowed in public catering places, at airports, rail, road and water stations, on trains, and on ships for sea and river transport. The Code of Administrative Violations includes fines and penalties for violation of the smoking ban.

Overall, 33.0% (29.6, 36.6%–95% CI) of adults who visited various public places (government buildings, private workplaces, health care facilities, schools, colleges or universities, restaurants, bars or nightclubs, cafes or cafeterias, and public transportation) in the past 30 d were exposed to tobacco smoke. Overall, 19.0% of adults who worked indoors were exposed to tobacco smoke, and among non-smokers, the rate was 13.4% (10.7, 16.7%–95% CI). Therefore, despite the ban on smoking in public places, adults were still exposed to tobacco smoke at work and in public places. It was due to limitations of partial smoke-free laws showing a need for existing law enforcement.

People who quit smoking significantly reduced their risk of disease and premature death (4,5). Article 14 of WHO FCTC addresses the demand for reduction measures to combat tobacco dependence and support cessation. Many smokers want to stop smoking and need encouragement and support offered through counseling and medications (4).

In Kazakhstan, chronic non-communicable disease screening procedures for the target population require practitioners to obtain information about the smoking status of a patient. Anti-tobacco resources are available in public primary health care settings. Cost-covered nicotine-replacement therapy and national tobacco quit line are not generally available. Almost a third (29.5%) of smokers had attempted to quit tobacco in the past 12 months. Along with improvements in asking patients whether they smoke or not, and providing cessation advice, improvements need to be made to increase the awareness of health care providers in terms of smoking cessation counseling and other quit-support services.

Article 11 of WHO FCTC states that the adoption and implementation of effective measures should include the placement of warning labels on cigarette packages. The Guidelines for the Implementation of Article 11 of the WHO Framework Convention on Tobacco Control supports the fact that combined written and graphic health messages on the packaging of tobacco products are more likely to attract the public’s attention and raise awareness on the health effects of tobacco than text warnings alone (5).

According to Article 12 of FCTC, every party has an obligation to support and promote public awareness on tobacco control issues, health effects of tobacco use and exposure to tobacco smoke, and the health benefits of quitting tobacco use. Active and continuous anti-tobacco campaigns increase people’s awareness about the harms of tobacco use, prompting the reduction in tobacco use and exposure to tobacco smoke, and increases the rates of smokers who quit (5). Knowledge of health consequences of smoking and secondhand smoke exposure among men was considerably lower than among women. The level of knowledge about smoking health risks was also lower among current smokers than the general population. Since 2013 in Kazakhstan, pictorial health warnings have been placed on cigarette packages and, along with text warnings, these occupy 40% of each large side of the cigarette package. Almost two-thirds of current smokers (58.0%) were considering quitting because of the newly introduced pictorial health warning labels on cigarette packages.

Article 13 of FCTC calls for a complete ban on tobacco advertising, promotion, and sponsorship. The total ban for any kind of tobacco marketing can reduce tobacco consumption and protect the youth from smoking initiation (4,12).

Kazakhstan has a general ban on tobacco and tobacco products advertising, which extends to sponsorship and promotion too. Despite the ban, GATS showed that respondents (25.7%) had been exposed to some type of cigarette advertisement, sponsorship, or promotion. Stores, where cigarettes were sold, were a key site of cigarette marketing.

Article 6 of the FCTC addresses the importance of price policy for and tax on tobacco products. An increase in cigarette tax reduces the rates of adults and youths who smoke. A 10% increase in the real price of cigarettes may cause a 3 to 5% drop in cigarette consumption in high-income countries, and an 8% reduction in low-and-middle-income countries (4,13,14).

Kazakhstan applies a specific excise tax. Differential rates apply (operating in a tier system) for filter and non-filter cigarettes. In 2014, the excise tax on manufactured cigarettes increased by 93.5% of the products, and since then, the annual increase in the excise tax has been 30%, up until 2016. Nevertheless, according to the results of GATS Kazakhstan 2014, cigarettes remain largely affordable for the population.

The key survey limitation is related to self-reported responses of the respondents with probability of under- or over-estimation of their behavioral status or knowledge of tobacco-related situation.

## Conclusion

The first GATS Kazakhstan results provide valid and comparable data on adult tobacco use, which was previously absent for our middle-income country. People remain exposed to secondhand smoke at work and in public places, despite the existence of a law that partially prohibits smoking in certain public spaces. The recently introduced graphical warnings on cigarette packs have been noticed by nearly all smokers, and are effective in making smokers think about quitting. However, cigarettes are still widely available, and the population, especially at the point of sale, notices various forms of tobacco marketing. The results demonstrate new opportunities for research findings to transition into policy actions in Kazakhstan.

## Ethical considerations

Ethical issues (Including plagiarism, informed consent, misconduct, data fabrication and/or falsification, double publication and/or submission, redundancy, etc.) have been completely observed by the authors.
